# B-Lines by Lung Ultrasound Can Predict Worsening Heart Failure in Acute Myocardial Infarction During Hospitalization and Short-Term Follow-Up

**DOI:** 10.3389/fcvm.2022.895133

**Published:** 2022-05-02

**Authors:** Jiexin He, Shixin Yi, Yingling Zhou, Xiangming Hu, Ziheng Lun, Haojian Dong, Ying Zhang

**Affiliations:** Department of Cardiology, Guangdong Cardiovascular Institute, Guangdong Provincial People's Hospital, Guangdong Academy of Medical Sciences, Guangzhou, China

**Keywords:** lung ultrasound, acute myocardial infarction, B-lines, pulmonary edema, heart failure

## Abstract

**Background:**

Acute myocardial infarction (AMI) with pulmonary edema shows a worse prognosis. Lung ultrasound (LUS) is a new tool for evaluating subclinical pulmonary congestion. It has been proved to predict prognosis in heart failure; however, whether it can be used as a short-term prognostic marker in AMI and provide incremental value to Killip classification is unknown.

**Methods:**

We performed echocardiography and LUS by the 8-zone method in patients enrolled in Guangdong Provincial People's Hospital undergoing percutaneous coronary intervention for AMI from March to July 2021. The lung water detected by LUS was defined as B-lines, and the sum of the B-line number from 8 chest zones was calculated. Besides, the classification into LUS according to the pulmonary edema severity was as follows: normal (B-line numbers <5), mild (B-line numbers ≥5 and <15), moderate (B-line numbers ≥15 and <30), and severe (B-line numbers ≥30). The NT-proBNP analysis was performed on the same day. All patients were followed up for 30 days after discharge. The adverse events were defined as all-cause death, worsening heart failure in hospitalization, or re-hospitalization for heart failure during the follow-up.

**Results:**

Sixty three patients were enrolled consecutively and followed up for 30 days. The number of B-lines at admission (median 7[3–15]) was correlated with NT-proBNP (*r* = 0.37, *p* = 0.003) and negatively correlated with ejection fraction (*r* = −0.43; *p* < 0.001) separately. In the multivariate analysis, B-line number was an independent predictor of short-term outcomes in AMI patients (in-hospital, adjusted OR 1.13 [95% CI: 1.04–1.23], *P* = 0.006; 30-day follow-up, adjusted OR 1.09 [95% CI: 1.01–1.18], *P* = 0.020). For in-hospital results, the area under the receiver operating characteristic curves (AUCs) were 0.639 (*P* = 0.093), 0.837 (*P* < 0.001), and 0.847 (*P* < 0.001) for Killip, LUS and their combination, respectively. For the diagnosis of 30-day adverse events, the AUCs were 0.665 for the Killip classification (*P* = 0.061), 0.728 for LUS (*P* = 0.010), and 0.778 for their combination (*P* = 0.002).

**Conclusion:**

B-lines by lung ultrasound can be an independent predictor of worsening heart failure in AMI during hospitalization and short-term follow-up and provides significant incremental prognostic value to Killip classification.

## Introduction

Myocardial infarction is an acute syndrome caused by the sudden blockage of the coronary arteries. Even though the coronary arteries have been opened in time, the prognosis remains poor in some patients. Acute myocardial infarction (AMI) complicated by heart failure is considered the main cause of increased mortality. Early risk stratification is essential for the postoperative management of AMI (1).

Killip Classification was initially described in 1967 and extensively used in the risk stratification of AMI patients, as it was considered to have a significant prognostic value. However, lung auscultation has shown poor sensitivity and accuracy in detecting mild pulmonary edema, which could decrease the accuracy of Killip classification (2).

Conversely, lung ultrasound (LUS) has been gaining attention over the past decade as a non-invasive tool for the detection and quantification of pulmonary congestion in both ambulatory and hospitalized patients with heart failure (HF) (3). Sonographic assessment of extravascular lung water is based on reverberation artifacts from the pleural line, which are thought to originate from the interlobular septa thickened by fluid. These discrete laser-like vertical hyperechoic reverberations are known as “B-lines” (also “comet tails” or “lung comets”) (4). Therefore, LUS is sensitive to the water deposited in the lung, potentially playing an important role in monitoring pulmonary congestion during hospitalization and improving risk assessment.

Therefore, indications for LUS are growing in cardiology, especially in patients with heart failure or dyspnea (5, 6). LUS can predict the prognosis of patients with acute and chronic heart failure (7, 8), and LUS-guided treatment can reduce the rehospitalization rate of patients with HF (9). A recent study suggested that LUS added to the Killip classification was more sensitive than physical examination to identify patients with ST-Elevation Myocardial Infarction (STEMI) at risk of in-hospital mortality (10). Furthermore, another study found that B-lines can help predict HF in patients with acute myocardial infarction during hospitalization (11). However, whether B-lines can predict the short-term prognosis of AMI after discharge is unknown. We aimed to evaluate the short-term prognostic ability of LUS in patients with AMI.

## Materials and Methods

### Study Design and Participants

The prospective cohort enrolled 63 consecutive patients admitted with an AMI diagnosis (with or without ST-segment elevation) to the Emergency Department of Guangdong Provincial People's Hospital from March to July 2021. Those who did not meet the inclusion criteria were excluded from the study. Inclusion criteria were: (i) age >18 years; and (ii) suspected AMI diagnosis, based on the presence of typical symptoms associated with ischemic abnormalities in the electrocardiogram, fulfilling the diagnostic criteria for AMI according to current guidelines (1). Exclusion criteria were: (i) pulmonary fibrosis or other severe diseases hampering image acquisition (significant pleural effusion, severe emphysema, pulmonary cancer, and so on), and absence of Killip classification or LUS at admission; (ii) non-obstructive myocardial infarction; or (iii) pregnancy. The research was approved by the Ethics Committee of Guangdong Provincial People's Hospital. Written informed consent was obtained from all patients.

All patients were treated with optimal medical therapy according to current guidelines (12, 13), and appropriate percutaneous coronary intervention (PCI) technical strategies were performed in time.

### Echocardiography and Lung Ultrasound

Patients underwent echocardiography and LUS as soon as possible on admission. Bedside transthoracic echocardiography and LUS were performed using Philips 7C ultrasound equipment with a 2.5 MHz phased array transducer. Left ventricle (LV) volumes and ejection fraction (EF) were obtained by two-chamber and four-chamber views using the biplane method of disk summation (modified Simpson's rule), according to the recommendations of the American Society of Echocardiography. The anteroposterior diameter of the left atrium (LA) can be measured in the parasternal long-axis view at the level of the aortic sinuses by using the leading-edge to the leading-edge convention. As recommended, measurements of tricuspid annular plane systolic excursion (TAPSE) were obtained. Diastolic function was assessed from the mitral inflow pattern by pulsed Doppler and tissue Doppler imaging to obtain the E/e' ratio (14).

LUS was performed at the same time as the echocardiography, and patients were placed in the supine position. The LUS examination was performed by the 8-zone method, and the number of B-lines of each zone was counted (15). We summed the total number of B-lines in 8 zones, and the classification into LUS according to the pulmonary edema severity was as follows: normal (B-line numbers <5), mild (B-line numbers ≥5 and <15), moderate (B-line numbers ≥15 and <30), and severe (B-line numbers ≥30) (16). All examinations were performed by one operator, unaware of laboratory data and clinical results.

### Killip Classification

Patients were evaluated by an experienced cardiologist at the emergency department to assess the signs and symptoms of clinical congestion, and the Killip classification was provided blinded to the results of the lung ultrasound. The classification into Killip I–IV was as follows: Killip I, no evidence of heart failure; Killip II, signs indicating a mild to moderate degree of heart failure (S3 gallop, rales half way up the lung fields, or elevated jugular venous pressure); Killip III, acute pulmonary edema (bilateral rales in more than half of both lung fields and dyspnea at rest), and Killip IV, cardiogenic shock (systolic blood pressure <90 mmHg and signs of poor perfusion) (17).

### Biochemical Analysis

All peripheral venous blood samples were taken on admission and discharged into sterile tubes containing Ethylene Diamine Tetraacetic Acid (EDTA). N-terminal pro-brain natriuretic peptide (NT-proBNP) analysis was performed using the Abbott Architect assay (Abbott Diagnostics, Abbott Park, IL, USA). In addition, troponin T (TnT) was measured using the electrochemiluminescence method (Roche Diagnostics), and soluble ST2(sST2) was assessed using the Presage ST2 Assay (Waltham, MA).

### Follow-Up and Outcomes

All patients were followed up for 30 days after discharge. The adverse events were defined as all-cause death, worsening heart failure in hospitalization (required intravenous diuretic treatment or diuretic increase), or re-hospitalization during the follow-up. The electronic medical records in the hospital were reviewed, and the follow-up data were obtained by telephone interviews and clinic visits from discharge. The coronary angiography data that the number of obstructed coronary vessels observed during the procedure was also recorded.

### Statistical Analysis

Continuous variables were expressed as mean (± SD) or median (25th−75th percentiles), as appropriate. Categorical variables were presented as counts and percentages. The B-line number was analyzed as a continuous variable (total B-line numbers) and a categorical variable (LUS classification, the four grades defined above). The correlation between the total B-line numbers, NT-proBNP, and EF was assessed with a non-parametric Spearman correlation coefficient analysis.

Logistic regression models (unadjusted and adjusted) were used to assess the continuous association between B-lines and short-term outcomes. Models were adjusted for potential confounding variables using a forward-conditional selection procedure, including age, Killip classification, log-transformed NT-proBNP concentration, etc. These covariates were chosen based on their clinical importance concerning the outcome, using a limited number of variables to prevent overfitting.

Kendall's tau correlation was used to assess the relationship between Killip and LUS classification. The ROC curve was analyzed to evaluate the efficacy of the different variables, and the areas under the curve (AUCs) were calculated. Two-sided significance levels of 0.05 were used for all analyses. Data were analyzed using SPSS (version 25.0.0; IBM Company).

## Results

### Patient Characteristics

The main clinical data of the 63 patients included are reported in Table 1. 79% of patients were male in the whole cohort, with an average age of 63 ± 12 years and a median EF of 47% (38–54%). Furthermore, 70% of patients had STEMI, and 79% had multiple vessel lesions. A few patients had atrial fibrillation (6%) or chronic kidney disease (2%), which may be the confounding factor that affected B-lines or NT-proBNP concentration.

**Table 1:** Patient characteristics (*N* = 63).

**Characteristic**	**Overall (*n* = 63)**
Age (years)	63 ± 12
Men	50 (79%)
STEMI	44 (70%)
**Medical history**	
Hypertension	30 (48%)
Diabetes	25 (40%)
Atrial fibrillation	4 (6%)
Chronic kidney disease	1 (2%)
Smoking (previous or current)	25 (40%)
Previous PCI	13 (21%)
**Admission characteristics**	
Systolic blood pressure (mmHg)	124 ± 23
Diastolic blood pressure (mmHg)	77 ± 16
dyspnea	14 (22%)
Ankle edema	1 (2%)
Acute pulmonary inflammation	2 (3%)
Cardiac shock	8 (13%)
**Killip classification**	
I	38 (60%)
II	12 (19%)
III	6 (10%)
IV	7 (11%)
**Number of vessels**	
Single vessel	11 (18%)
Multiple vessels	50 (79%)
**B-lines**	7 (3-15)
Normal	2 (1-4)
Mild	8 (7-13)
Moderate	16 (11-18)
Severe	23 (18-27)
**Laboratory results**	
NT-proBNP (pg/ml)	4,793 (803–4939)
TnT (ng/ml)	3,103 (719–4767)
sST2 (ng/ml)	40 (26–40)
Creatinine (mg/dl)	97 (66–102)
Albumin (g/dl)	36 (34–38)
**Echocardiography**	
EF(%)	47 (38–54)
LA diameter (mm)	35 ± 5
E/e' ratio	15 ± 6
TAPSE (mm)	21 ± 3

*STEMI, ST-segment elevation myocardial infarction; PCI, percutaneous coronary Intervention; LUS, lung ultrasound; NT-proBNP, N-terminal pro-brain natriuretic peptide; TnT, troponin T; sST2, soluble ST2; EF, ejection fraction; LA, left atrium; TAPSE, Tricuspid Annular Plane Systolic Excursion*.

When patients arrived in the emergency room, 13% had a cardiac shock, only 22% had dyspnea, and <5% showed ankle edema. Overall, 60% of patients were classified as Killip I class. However, the median B-lines at admission were 7 (IQR, 3–15), indicating that most of them had mild pulmonary edema at least. Patients also had high levels of NT-proBNP, sST2, and TnT. Echocardiography showed mild dilation in the left atrium, and the E/e' ratio increased (Table 1).

### B-Lines Acted as an Independent Predictor of Short-Term Adverse Events

The number of B-lines correlated with NT-proBNP values (*r* = 0.37, *p* = 0.003) and negatively correlated with EF measured by echocardiography (*r* = −0.43; *p* < 0.001), which are usually used to predict heart failure and poor prognosis. In our sample, 17 patients (27%) and 15 patients (24%) developed adverse events during hospitalization and 30 days of follow-up, respectively. During hospitalization, 3 (5%) died from all causes, and 14 (22%) patients developed worsening HF, while during the 30 days follow-up, 3 (5%) died, 9 (14%) developed worsening HF, and 3(5%) were readmitted.

**Figure 1:**
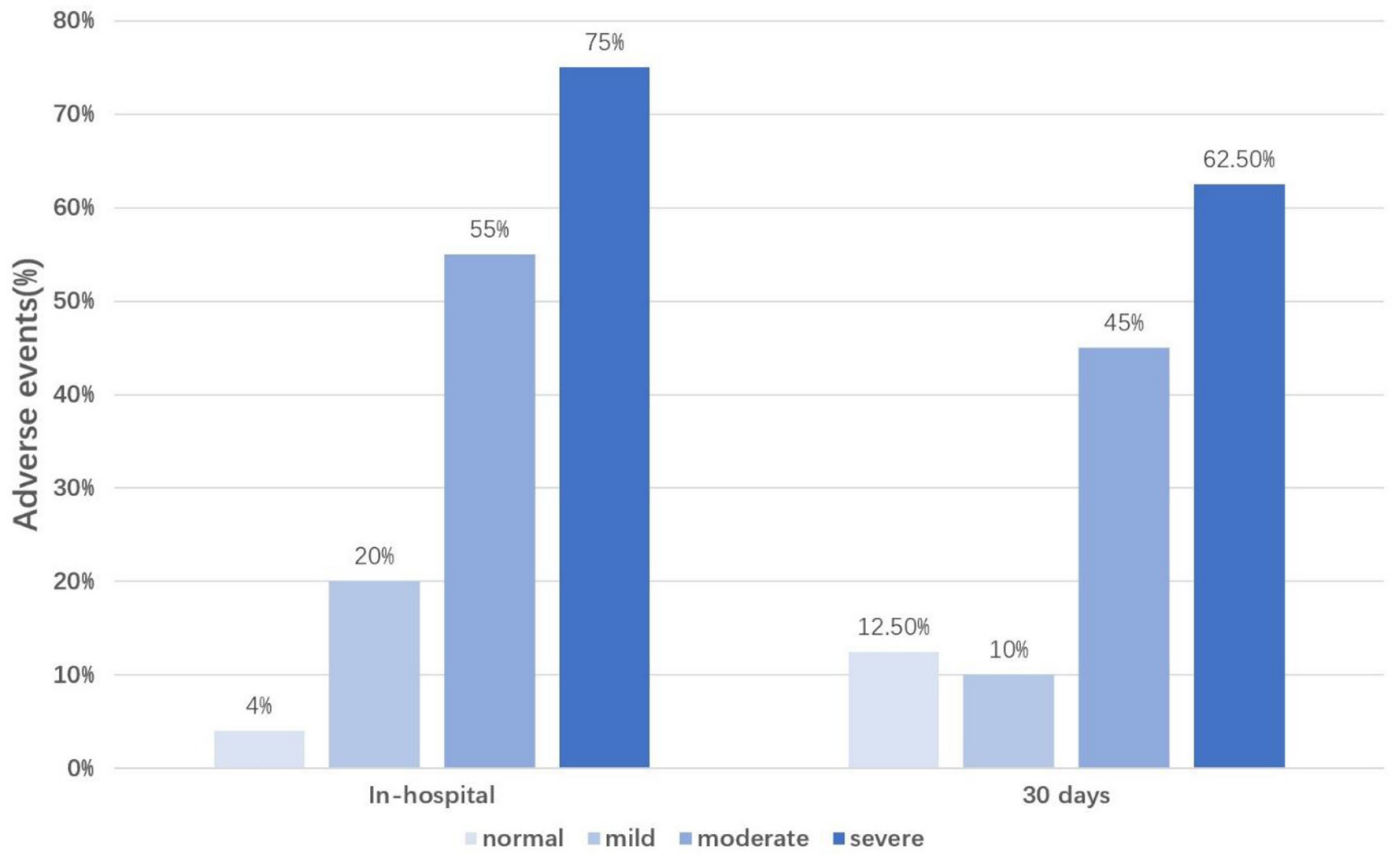
Percentage of adverse events occurrence according to the LUS classification. LUS classification, the total number of B-lines was divided into four groups according to the severity of pulmonary edema (normal, B-line numbers <5; mild, B-line numbers ≥5 and <15; moderate, B-line numbers ≥15 and <30; severe, B-line numbers ≥30); In-hospital, during the hospitalization; 30 days, during 30 days follow-up.

Compared to patients with dry lungs or mild pulmonary edema, adverse cardiovascular events were more significantly increased in patients with moderate to severe pulmonary edema (Figure 1).

We constructed an unadjusted and adjusted logistic regression model to identify the factors that could predict the short-term outcomes of AMI among the included co-variables; results are shown in Tables 2, 3. For in-hospital events, on univariate analysis, age (OR 1.08 [95% CI: 1.02–1.14], *P* = 0.006), total B-line numbers (OR 1.14 [95% CI:1.05–1.24], *P* = 0.002), creatinine (OR 1.02 [95% CI: 1.01–1.04], *P* = 0.040), Killip II-IV (OR 2.95 [95% CI: 0.94–9.29], *P* = 0.041), Log NT-proBNP (OR 4.14 [95% CI:1.29–13.32], *P* = 0.017), EF (OR 0.94[95% CI:0.89–0.99], *P* = 0.027), and LA diameter (OR 1.16 [95% CI:1.02–1.32], *P* = 0.026) all had statistical significance, but only the first three contributed independent information in a multivariable model.

**Table 2:** Univariate analysis for in-hospital and 30 days composite outcome.

**Characteristic**	**In-hospital**		**30 days**	
	**OR (95%CI)**	***P*-value**	**OR (95%CI)**	***P*-value**
Age (years)	1.08 (1.02–1.14)	0.006^*^	1.05 (0.99–1.11)	0.070
STEMI	4.40 (0.89–21.60)	0.070	1.04 (0.27–3.98)	0.950
Diabetes	2.11 (0.68–6.53)	0.200	1.31 (0.40–4.34)	0.660
Shock	3.23 (0.71–14.76)	0.130	3.08 (0.55–17.35)	0.200
Previous PCI	1.98 (0.54–7.21)	0.300	6.00 (1.39–25.86)	0.016^*^
Killip II-IV	2.95 (0.94–9.29)	0.041^*^	3.50 (1.02–12.03)	0.047^*^
Number of vessels	5.12 (0.66–39.58)	0.120	2.19 (0.46–10.45)	0.330
Total B-line numbers	1.14 (1.05–1.24)	0.002^*^	1.10 (1.02–1.18)	0.020^*^
Log NT-proBNP	4.14 (1.29–13.32)	0.017^*^	3.20 (1.03–9.94)	0.044^*^
Log TNT	3.34 (0.99–11.21)	0.050	1.80 (0.62–5.17)	0.280
Creatinine (mg/dl)	1.02 (1.01–1.04)	0.040^*^	1.01 (0.99–1.03)	0.107
EF (%)	0.94 (0.89–0.99)	0.027^*^	0.92 (0.86–0.98)	0.008^*^
LA (mm)	1.16 (1.02–1.32)	0.026^*^	1.11 (0.97–1.26)	0.130
E/e' ratio	1.06 (0.97–1.16)	0.220	1.04 (0.94–1.13)	0.470

*STEMI, ST-segment elevation myocardial infarction; PCI, percutaneous coronary Intervention; Log NT-proBNP, log-transformed N-terminal pro-brain natriuretic peptide; Log TNT, log-transformed troponin T; EF, ejection fraction; LA, left atrium; OR, odds ratio*.

* *P < 0.05*.

*Statistically significant results are marked in bold*.

**Table 3:** Multivariable analysis for in-hospital and 30 days composite outcome.

**Characteristic**	**In-hospital**		**30 days**	
	**OR (95%CI)**	***P*-value**		**OR (95%CI)**	***P*-value**
Age (years)	1.08 (1.01–1.14)	0.020^*^		1.04 (0.97–1.12)	0.280
Total B-line numbers	1.13 (1.04–1.23)	0.006^*^		1.09 (1.01–1.18)	0.020^*^
Killip IV	17.52 (0.89–344.33)	0.060		2.82 (0.27–29.45)	0.390
Number of vessels	3.17 (0.23–42.85)	0.390		0.83 (0.14–5.05)	0.840
Log NT-proBNP	1.17 (0.15–9.23)	0.880		1.15 (0.24–5.58)	0.870
Creatinine (mg/dl)	1.03 (1.01–1.07)	0.037^*^		1.01 (0.99–1.03)	0.300

*Log NT-proBNP, log-transformed N-terminal pro-brain natriuretic peptide; OR, odds ratio; In-hospital means outcomes during the hospitalization; 30 days means outcomes during 30 days after discharge*.

* *P < 0.05*.

*Statistically significant results are marked in bold*.

For the 30-day follow-up, the univariate analysis showed that previous PCI (OR 6.00 [95% CI: 1.39–25.86], *P* = 0.016), total B-line numbers (OR 1.10 [95% CI: 1.02–1.18], *P* = 0.020), Killip II-IV (OR 3.50 [95% CI: 1.02–12.03], *P* = 0.047), Log NT-proBNP (OR 3.20 [95% CI: 1.03–9.94], *P* = 0.044), and EF (OR 0.92 [95% CI: 0.86–0.98], *P* = 0.008) predicted outcome at 30 days. However, only total B-line numbers contributed independent information in a multivariable model. The association between total B-line numbers and the outcome was similar during the in-hospital period (adjusted OR 1.13 [95% CI: 1.04–1.23], *P* = 0.006) and 30 days after discharge (adjusted OR 1.09 [95% CI: 1.01–1.18], *P* = 0.020). Therefore, B-lines can be a stable and independent predictor of short-term adverse events in AMI patients.

### Correlation Between LUS and Killip Classification

**Figure 2:**
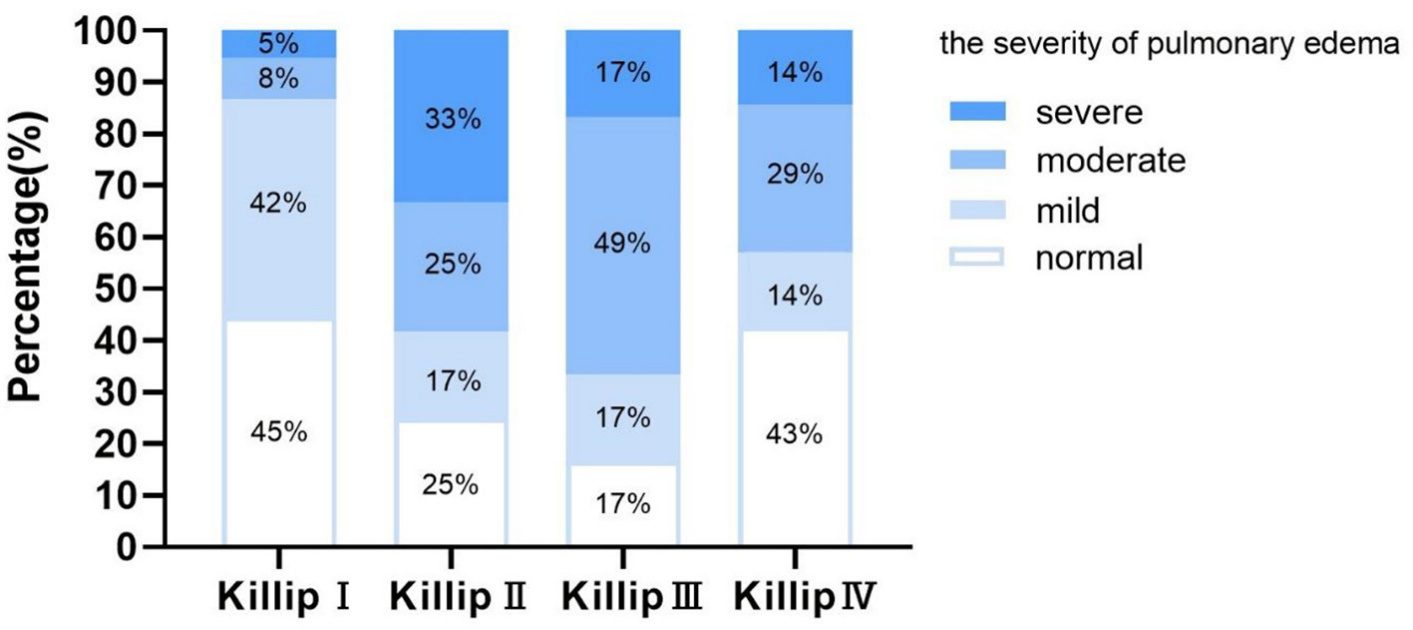
Proportion of the severity of lung water in patients with Killip I–IV. Normal, B-line numbers <5; Mild, B-line numbers ≥5 and <15; Moderate, B-line numbers ≥15 and <30; Severe, B-line numbers ≥30.

In different Killip grades, the severity of pulmonary edema showed a large variation (Figure 2). In patients with Killip I, normal and mild pulmonary edema accounted for up to 87%. However, with the development of heart failure, the proportion of patients with moderate and severe pulmonary congestion increased significantly. Among the patients with Killip II, 58% had at least moderate lung water, while in Killip III, 66% had at least moderate lung water. Unexpectedly, in patients with Killip IV, 43% were normal, while severe edema only accounted for 14% of patients. Kendall's tau correlation analysis showed a correlation between LUS and Killip classification (κ = 0.26, *P* = 0.018). A similar correlation was also found between the total B-line numbers and Killip classification (κ = 0.27, *P* = 0.008).

### Comparison of LUS and Killip Classification to Predict Adverse Events

**Figure 3:**
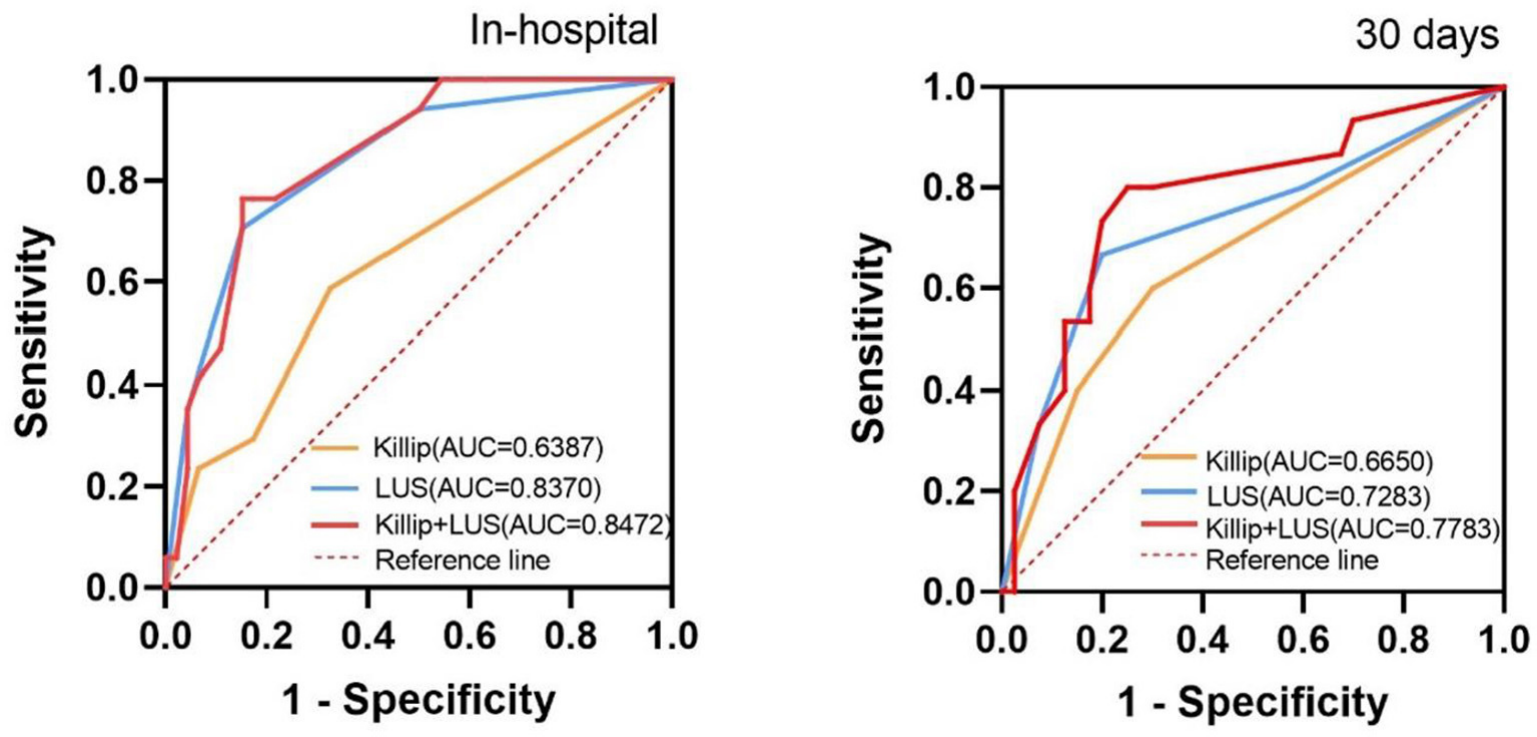
Receiver operating characteristic curves for “Killip”, “LUS”, and “Killip+LUS” classification to predict the in-hospital and 30 days composite outcome. AUC, the area under the curve. In-hospital, during the hospitalization; 30 days, during 30 days follow-up; Killip, Killip classification; LUS, LUS classification; Killip+LUS, the combination of LUS and Killip classification.

The ability of the LUS and Killip classification to diagnose the short-term clinical outcomes of AMI was analyzed using ROC curve analysis. The area under the ROC curve for the prediction of in-hospital adverse events was 0.639 for the Killip classification (95% CI 0.48–0.80, *P* = 0.093), 0.837 for the LUS classification (95% CI 0.73–0.95, *P* < 0.001), and 0.847 for the combination of LUS and Killip classification (95% CI 0.75–0.95, *P* < 0.001). Moreover, for the diagnosis of 30-day cardiovascular adverse events, the AUCs were 0.665 for the Killip classification (95% CI 0.50–0.83, *P* = 0.061), 0.728 for LUS classification (95%CI 0.56–0.89, *P* = 0.010), and 0.778 for the combination of LUS and Killip classification (95%CI 0.63–0.92, *P* = 0.002). These two ROC curves showed that the combination of LUS and Killip classification was superior to Killip classification alone in predicting short-term adverse outcomes in AMI (Figure 3).

## Discussion

In a prospective cohort of patients with AMI undergoing PCI, we found that B-lines by lung ultrasound can be an independent predictor of adverse cardiovascular events during hospitalization and 30 days of follow-up. AMI patients with more severe pulmonary edema may have a higher risk of adverse events. According to the ROC curves, the combination of LUS and Killip had a superior predictive value for evaluating the short-term outcomes in AMI. It has been proved that LUS can provide significant incremental prognostic value to Killip classification.

Although some studies have evaluated the in-hospital mortality of patients with myocardial infarction by LUS, this is the first study to further evaluate the short-term prognostic value of B-lines.

It is well-known that AMI patients often face complication related to acute left heart failure, which is associated with a higher risk of short-term mortality compared to AMI alone (18). Pulmonary congestion is a prominent element in HF. However, lung auscultation has limited sensitivity and specificity (19). Pulmonary congestion is likely to be ignored, and prompt diuretic treatment cannot be provided. Most patients had at least mild pulmonary edema in this study, but 60% were diagnosed with Killip I. Therefore, edema may have been underestimated due to some false negatives in the Killip grades.

LUS is becoming an effective method for detecting interstitial pulmonary edema (15). Although the LUS protocol has not been unified thus far, we chose the simplified 8-zone method because it was considered effective and less time-consuming. In our study, a positive correlation was found between the number of B-lines and NT-proBNP level, and a negative correlation was found in EF. Our results indicated that B-lines by lung ultrasound could be a promising marker to predict heart failure, such as secreted frizzled-related protein 5(SFRP5) and extracellular volume fraction by cardiovascular magnetic resonance (20, 21). Alberto Palazzuoli et al. also confirmed that B-lines are strongly associated with clinical assessment, biomarkers, or echocardiography. B-lines at discharge add important information regarding risk stratification in acute heart failure (AHF) patients.

In addition to heart failure, recent studies have begun to focus on the prognostic value of B-lines for AMI. AMI is usually complicated with LV afterload and filling pressures increasing, leading to pulmonary congestion. However, In Gustavo N. Araujo et al.'s study, inflammation and vascular permeability were also contributed to extravascular lung water increase in AMI patients. Bedetti et al. found that LUS added additional prognostic value to the Global Registry in Acute Coronary Events score in 470 patients admitted with acute coronary syndromes (22). Jorge et al. also found that LUS performed at admission can help to predict heart failure in patients with AMI (11). In our sample, among patients without lung water, only 4% (during hospitalization) and 12.5% (during 30-day follow-up) had adverse events, indicating that negative results of LUS are more likely to indicate a good prognosis for patients with AMI. However, with the gradual aggravation of pulmonary edema, the proportion of patients with adverse events gradually increased, suggesting that we may need to pay more attention to patients with AMI combined with moderate to severe pulmonary edema.

Our results confirmed that there was a correlation between LUS and Killip classification. Although the severity of pulmonary edema assessed by LUS was not as severe as expected in Killip IV patients, this might have occurred because they were more usually treated with non-invasive or invasive ventilation, which could reduce lung water before LUS examination (23). Killip classification relies mainly on lung rales to determine congestion at admission; however, as mentioned above, auscultation results can be false negatives. Araujo et al. suggested that admission LUS added to the Killip classification was a feasible and more sensitive method of identifying patients with STEMI at risk for in-hospital outcomes than physical examination (10). Moreover, our ROC curves also support this notion and add some new information: the combination of LUS and Killip was superior to Killip alone for predicting short-term adverse outcomes in AMI, in hospital and during 30 days of short-term follow-up.

Lastly, in univariate and multivariate regression analyses, B-lines can be used as a stable independent predictor of the short-term adverse events of AMI. The correlation between the total B-line numbers and outcomes during hospitalization was slightly higher than during the 30-day follow-up. Platz et al. (3) assessed the prognostic importance of B-lines in acute heart failure (AHF). They found that the relationship between B-lines and outcomes was stronger closer to hospital discharge and diminished over time, consistent with our results. This may be because pulmonary edema caused by AHF is usually transient. It may be feasible to conduct large clinical trials to evaluate whether LUS-guided therapy can improve the prognosis of AMI patients.

### Limitation

This was a single-center cohort study with small sample size, limiting our results' generalizability and rendering the estimation of their associations imprecise. In addition, the follow-up period was relatively short. Further extensive independent studies are warranted to confirm the strong association observed between B-line numbers and AMI patients' risk stratification.

In addition, due to the urgent condition of certain AMI patients, they did not have time to perform LUS before PCI. However, they all completed LUS within 24 h of the perioperative period, which may have had a minor effect on our data and results (24). Lastly, since patients are admitted supine, height and weight could not be measured, and BMI cannot be calculated, which may have affected the calibration of some indicators.

## Conclusion

In a prospective cohort of patients with AMI undergoing PCI, we found that B-lines by lung ultrasound can be an independent predictor of worsening heart failure in AMI during hospitalization and short-term follow-up. Besides, B-line numbers can provide significant incremental prognostic value to Killip classification.

## Data Availability Statement

The raw data supporting the conclusions of this article will be made available by the authors, without undue reservation.

## Ethics Statement

The studies involving human participants were reviewed and approved by the Ethics Committee of Guangdong Provincial People's Hospital. The patients/participants provided their written informed consent to participate in this study.

## Author Contributions

Material preparation, data collection, and analysis were performed by JH, SY, and ZL. The first draft of the manuscript was written by JH and all authors commented on previous versions of the manuscript. YZ and HD are equally accountable for all aspects of the work in ensuring that questions related to the accuracy or integrity of any part of the work are appropriately investigated and resolved. All authors contributed to the study conception and design and read and approved the final manuscript.

## Funding

This work was supported by the Science and Technology Project of Guangzhou City (202102080223).

## Conflict of Interest

The authors declare that the research was conducted in the absence of any commercial or financial relationships that could be construed as a potential conflict of interest.

## Publisher's Note

All claims expressed in this article are solely those of the authors and do not necessarily represent those of their affiliated organizations, or those of the publisher, the editors and the reviewers. Any product that may be evaluated in this article, or claim that may be made by its manufacturer, is not guaranteed or endorsed by the publisher.
